# Socio-demographic characteristics of a First-Episode Psychosis Programme

**DOI:** 10.1192/j.eurpsy.2025.2258

**Published:** 2025-08-26

**Authors:** C. Rodriguez Valbuena, M. A. Andreo Vidal, M. Calvo Valcárcel

**Affiliations:** 1 Psychiatry, Hospital Clínico Universitario de Valladolidd, Valladolid, Spain

## Abstract

**Introduction:**

While it is known that of those people who experience psychotic experiences, approximately 75% of them do so for the first time between the ages of 15 and 30 and that the majority are male, there has recently been increasing interest in the incidence of psychosis in other population groups.

**Objectives:**

The present study aims to analyse the sociodemographic data collected over a period of ten months in a First-Episode Psychosis Programme in a third level hospital, such as the Hospital Clínico Universitario de Valladolid.

**Methods:**

It is a retrospective observational study. Patients have been recruited during ten months and those who presented an episode of the psychosis spectrum for the first time (according to DSM-V diagnostic criteria) were included. Different socio-demographic data regarding their age, sex, marital status and employment status have been collected at the time of their inclusion in the programme.

**Results:**

A sample of 23 patients was recruited, of which 26% were women (n=6) and 74% were men (n=17).

The mean age was 29.95 years.

Regarding marital status, 70% of the patients in the sample were single (n=16), 17% were married (n=4), and 13% were living with a partner (n=3). There were no divorced or widowed patients.

In terms of employment, 36% (n=8) of the patients were in employment at the time of admission to the programme. 26% (n=6) were studying, 21% (n=5) were unemployed, 13% (n=3) were on sick leave and 4% (n=1) were receiving a pension.

**Conclusions:**

Socio-demographic data, in general, are as expected in a programme of these characteristics. However, it should be noted that the mean age of the patients recruited is above that most frequently described in the literature. However, we believe that it would be necessary to increase the sample size to be able to offer more robust results.

**Disclosure of Interest:**

None Declared

